# Strengthening the reporting of harms of all interventions in clinical trials

**DOI:** 10.5694/mja2.51755

**Published:** 2022-10-26

**Authors:** Christina Abdel Shaheed, Christopher G Maher, Ann‐Mason Furmage, Tammy Hoffmann, Andrew J McLachlan

**Affiliations:** ^1^ University of Sydney Sydney NSW; ^2^ Consumer Advisory Group Royal Prince Alfred Hospital Sydney NSW; ^3^ Institute for Evidence‐Based Healthcare Bond University Gold Coast QLD

**Keywords:** Adverse events, Adverse drug reactions, Clinical trials as topic

Better reporting of harms is needed in clinical trials to support better decision making

Harms in clinical trials may be characterised by adverse events (AEs) and adverse reactions. Both terms refer to untoward medical occurrences in a trial participant, but the second term presumes causation and is reserved for events directly attributed to the investigational treatment. Harms reporting in clinical trials is critical to understanding the nature and likelihood of experiencing unwanted effects resulting from or following treatment. Evidence to guide safe and effective use of treatments requires information on both possible benefits and harms. The CONSORT checklist for randomised controlled trials^1^ mandates reporting of harms, with specific guidance provided in the CONSORT extension for harms.^2^ However, a key problem is that information on harms is absent or reported inconsistently in clinical trials, warranting efforts to strengthen reporting of harms globally.

## Inadequate reporting may over‐ or underestimate harms

Unfortunately, in drug trials, AEs are measured and reported inconsistently, consideration of causality is often missing, and it is often unclear what AEs took place during the intervention versus the post‐intervention period.^3^, ^4^, ^5^ But the true risk of harms caused by treatment is largely unknown for three key reasons. First, randomised controlled trials are optimally designed to evaluate an efficacy endpoint; therefore, AE endpoints are typically underpowered or not adequately captured. Second, although a country's drug regulator may have specific reporting requirements around causality of harms,^6^ causality data are often excluded from the published report (ie, AEs are reported but not adverse reactions).^3^, ^4^, ^5^ This is particularly concerning when information on causality is missing for serious adverse events (SAEs) — “event/adverse reaction that results in death, is life‐threatening, requires hospitalisation or prolongation of existing hospitalisation, results in persistent or significant disability or incapacity, congenital anomaly”.^7^ Third, many drug trials report the cumulative frequency of AEs during both the intervention and follow‐up periods.^4^ Therefore, it is not possible to determine the frequency and type of AEs that took place during the intervention period, when an argument for a causal relationship (and temporal relationship based on likely exposure) is more plausible than in the follow‐up period, when the drug has left the system (eg, for drugs with a short half‐life). This can lead to an overestimation of drug‐related harms.

An under‐recognised problem is the inconsistent or lack of any AE reporting in clinical trials evaluating non‐drug interventions (eg, physical, psychological and surgical interventions).^5^, ^8^, ^9^, ^10^, ^11^, ^12^, ^13^, ^14^ This has been highlighted by recent large reviews, which reveal there are significant problems measuring and reporting AEs in non‐drug trials. In 2021, a review of 249 trials evaluating exercise for chronic low back pain found that only 12 trials measured any AEs in a systematic way.^8^ A review of trials of psychological interventions, funded by the National Institute for Health and Care Research, reported that none mentioned the occurrence of an AE in their final report and called for improvements in reporting practices;^14^ yet reporting problems persist.^15^ For example, in a 2022 review of psychological interventions for chronic back pain, only about 20% of the 97 trials included reported AEs, and most reported that no AEs occurred.^16^ These findings are unusual because when following people in a trial and measuring AEs carefully, it would be expected to see a proportion of these individuals reporting (or having experienced) some untoward medical event. In the PACE trial on the use of paracetamol for back pain, for example, about 20% of patients taking placebo reported experiencing an adverse event.^17^

Similarly, there are trials of behavioural interventions, for example, in children with attention deficit/hyperactivity disorder (ADHD) which did not report AEs of any type in the trial publication.^18^ Yet treatment emergent AEs, such as frustration, aggression and emotional reaction, are possible and have been previously documented with behavioural interventions in young children with ADHD.^19^

Study design features (eg, pragmatic design), and the prevailing belief that many non‐drug interventions are safe, have also been cited as reasons for no attention paid to recording or reporting AEs.^11^ For example, in the US replication of the STarTBack trial (stratified primary care management for low back pain with current best practice), the authors cited the trial's pragmatic nature and low risk of the intervention as reasons for not collecting AE data.^11^ Even in surgical trials, AEs are not always reported, and when they are reported, pooling of intra‐ and postoperative AEs can complicate interpretation.^5^

## Strategies to strengthen AE reporting

Reasons for under‐reporting of harms in many non‐drug trials include universal definitions of AEs which do not explicitly mention physical, psychological or surgical interventions, study design considerations such as poor AE data collection, and a lack of awareness of and adherence to reporting requirements.^11^, ^20^ This has helped to perpetuate the belief that non‐drug interventions are generally safe,^8^, ^9^, ^10^, ^11^, ^12^ and may encourage the use of non‐drug interventions for certain conditions in the absence of robust evidence around benefits and harms. Confusion regarding harms nomenclature is also likely contributing to the poor practices in reporting AEs.^21^ “Adverse events” describe untoward medical events that may or may not be related to the study intervention, but “adverse reactions” refer to untoward medical events related to the intervention. However, this convention is not followed in some fields, particularly where non‐drug interventions are being evaluated. For example, in the back pain field, a trial of physical therapy reported that AE data were not collected, although the study flowchart revealed there were 20 deaths.^11^

A key step to addressing the under‐reporting of harms in non‐drug trials is to raise awareness about these issues, educate about the difference in harms nomenclature, and improve transparency in clinical trial reporting, beginning from the trial registration and protocol stage through to providing the summary of results and other trial materials. In addition, the universal definition of AEs should be broadened to encompass all interventions. The scientific and clinical research community also has a responsibility to request more information from the study authors if information on harms is missing. However, this is time‐consuming and may not yield a response, reinforcing the importance of adequate primary reporting.

Although it is important to consider the feasibility (or lack thereof) of determining causality for non‐serious AEs, it should be necessary to report causality assessment outcomes for SAEs in published reports of clinical trials and incorporate this early in the trial protocol and registry. This would allow a more comprehensive understanding of the nature and frequency of SAEs that can be expected with any treatment. For drug trials, researchers should clearly distinguish between AEs occurring during and after treatment (ie, report them separately); and for surgical trials, AEs should be reported separately for the intra‐ and postoperative period. Furthermore, harms reporting should be consistent with conventional harms nomenclature. An event deemed to have a “reasonable possibility of a causal relationship”^7^ with an intervention should be described as reactions (eg, adverse reactions or serious adverse reactions) (Box).

Box 1 **Suggested reporting of harms based on trial type**

**Table:** 

Standard reporting strategy for all trials evaluating: ▶ medicinal drugs or medical devices (including complementary, alternative medicines) ▶ psychological or behavioural interventions (including digital therapeutics) ▶ surgical procedures ▶ physical therapies
Proportion of people experiencing, and frequency of, serious and non‐serious adverse events during and after the intervention (reported cumulatively and separately), as individuals may experience more than one adverse event at any one time. For surgical trials, this will be during the intra‐ and postoperative period
Causality for serious adverse events*
Causality for non‐serious adverse events, if feasible
Practical considerations: ▶ adverse event/serious adverse event data collection by a blinded investigator, where possible/appropriate ▶ for trials involving follow‐up at multiple time points (eg, > 1 year), avoid duplication of adverse event data collection and ensure the wording in the data collection tool or study questionnaire clearly avoids statements such as, “did you experience any adverse event since commencing the study versus since last completing the study questionnaire?”

* Adverse events or serious adverse events deemed to have a “reasonable possibility of a causal relationship”^7^ with an intervention should be clearly labelled as such in the trial report/publication (ie, adverse reaction or serious adverse reaction, respectively).

While it is expected that triallists undergo training such as Good Clinical Practice to familiarise themselves with best practice guidelines around harms measuring and reporting,^22^ the Human Research Ethics Committee (HREC) and the Data Safety Monitoring Board (DSMB) play a key role in ensuring that a trial has adequate measuring and reporting procedures for harms. Decisions need to be made early on, depending on the level of complexity of the trial and the intervention, about the suitability of appointing an independent, multidisciplinary DSMB. For many trials, particularly those involving medicinal products, medical devices, or surgical interventions, it is expected that a DSMB will be appointed to have oversight over the study's safety plan and reporting procedures.

The DSMB plays a critical role in mitigating the risks to data validity, trial credibility, and participant safety.^23^ Part of the core function of the DSMB is to make an independent assessment on the relatedness of SAEs (or where appropriate AEs) to the study intervention. This includes providing an evaluation of outcome (eg, recovered, recovered with sequelae, death, continuing AE or SAE) and providing recommendations to the investigator team on appropriate follow‐up actions (eg, when a trial participant becomes pregnant). Assessment of relatedness or causality will often require unblinding of the DSMB; however, where necessary, measures should be taken to maintain the blinding of the study investigators and those involved in data analysis. Practical considerations when forming a DSMB include avoidance of even numbers on a panel, in case adjudication is required to resolve disagreements.

Trial investigators also have a responsibility to the HREC to outline procedures for the handling and timelines for reporting SAEs. Importantly, there may be time‐critical reporting of SAEs to the sponsor, HREC and/or the country's regulatory body. This is the case in Australia, where for trials of medicinal drugs and devices, for example, investigators must inform the study sponsor about SAEs (requiring immediate reporting) within 24 hours of becoming aware of the event, which then triggers a cascade of further time‐critical reporting to the relevant regulator.^7^ The HREC must be satisfied that the procedures for measuring and reporting of harms adhere to best practice reporting guidelines and regulatory requirements.

Taken together, integrating these strategies into trial protocols, registries and, where applicable, extensions for CONSORT (eg, CONSORT for harms^2^ and CONSORT for non‐pharmacological studies^24^) may help researchers to report harms appropriately for all interventions. Such approaches may help reduce the under‐ or overestimation of the harms of treatments and guide better informed decision making by clinicians, patients and policymakers.

## Competing interests

No relevant disclosures.

## Provenance

Not commissioned; externally peer reviewed.

## Open access

Open access publishing facilitated by The University of Sydney, as part of the Wiley ‐ The University of Sydney agreement via the Council of Australian University Librarians.
